# Metabolomics analysis uncovers metabolic changes and remodeling of anti-VEGF therapy on macular edema

**DOI:** 10.1186/s40662-025-00444-2

**Published:** 2025-07-14

**Authors:** Congcong Yan, Quanyong Yi, Lina Ge, Ying Huang, Chun Yang, Bing Lin, Dan Jiang, Meng Zhou

**Affiliations:** 1 National Clinical Research Center for Ocular Diseases, Eye Hospital, Wenzhou Medical University, Wenzhou, 325027 China; 2 Ningbo Eye Hospital, Wenzhou Medical University, Ningbo, 315042 China

**Keywords:** Anti-VEGF, Aqueous humor, Macular edema, Metabolomics

## Abstract

**Background:**

Anti-angiogenic therapy with anti-vascular endothelial growth factor (anti-VEGF) is currently the first-line treatment for macular edema (ME), but the specific metabolic changes in the aqueous humor (AH) after intravitreal anti-VEGF injections remain poorly understood.

**Methods:**

A total of 120 AH samples from 60 ME patients before and after anti-VEGF treatment were collected from the ophthalmology clinic and ward of the Eye Hospital of Wenzhou Medical University. Non-targeted metabolomics analysis of the AH samples was performed using liquid chromatography-tandem mass spectrometry (LC–MS/MS). Orthogonal partial least squares discriminant analysis (OPLS-DA) was used to identify metabolite differences before and after anti-VEGF treatment in patients with different ME etiologies.

**Results:**

Distinct metabolomic profiles were observed between pre- and post-treatment samples. A total of 145 significantly altered metabolites were identified after anti-VEGF treatment, with 84 upregulated metabolites related to carbohydrate and amino acid metabolism, and 61 downregulated metabolites involved in amino acid metabolism. Both common and etiology-specific metabolic alterations were observed. In age-related macular degeneration (AMD)-ME, treatment-induced metabolic changes mainly involved amino acid metabolism, whereas in branch retinal vein occlusion (BRVO)-ME, lipid metabolism was primarily affected. Diabetic macular edema (DME) patients showed more complex metabolic alterations, involving amino acid, lipid and carbohydrate metabolism.

**Conclusions:**

Intravitreal anti-VEGF injections significantly alter AH metabolites in ME patients. These findings provide insight into underlying metabolic processes in ME pathogenesis and treatment efficacy.

**Supplementary Information:**

The online version contains supplementary material available at 10.1186/s40662-025-00444-2.

## Background

Age-related macular degeneration (AMD), branch retinal vein occlusion (BRVO) and diabetic macular edema (DME) are common retinal diseases that can lead to irreversible vision loss [1]. The first-line treatment for macular edema (ME) associated with AMD, BRVO, and diabetic retinopathy is anti-vascular endothelial growth factor (anti-VEGF) therapy. Vascular endothelial growth factors (VEGFs) play a critical role in the development of new blood vessels in the retina and choroid [2]. In addition, VEGFs increase the permeability of small blood vessels, which contributes to ME and other severe visual impairments, including retinal and vitreous hemorrhages, and elevated intraocular pressure [3]. Anti-VEGF treatments are effective in reducing vascular leakage by either directly blocking VEGF or its receptors, thereby aiding in the restoration of vision [4].

Several studies have investigated the changes in metabolites and proteins in the aqueous humor (AH) of ME patients compared to normal controls, as well as changes across different stages of ME [5–8]. While the benefits of anti-VEGF treatments in promoting revascularization and reducing vascular leakage are well recognized, the precise intraocular changes induced by these treatments remain poorly understood. The most common sight-threatening adverse events associated with anti-VEGF therapy include inflammation and increased intraocular pressure [9]. Other reported adverse events include lens damage, multiple retinal vein occlusions, cataract formation, hyphema, transient floaters, and visual loss [10]. Furthermore, the response to anti-VEGF therapy varies among patients. Tranos et al. observed that more than 50% of AMD patients show no improvement with standard anti-VEGF treatment, and approximately 10% were completely non-responsive, indicating resistance to therapy [11]. Although previous studies have investigated genomic and transcriptomic changes following anti-VEGF treatment [12–16], they do not fully capture the full biological complexity or the relationship between genotype and phenotype. Metabolites, as the products or intermediates of cellular processes, serve as a bridge among genotype, environment and phenotype, reflecting the functional state of biological systems [17]. Metabolic abnormalities are well-recognized hallmarks of diseases and are closely associated with therapeutic efficacy [18–21]. However, the specific metabolic alterations occurring within the AH of ME patients after intravitreal anti-VEGF injections are yet to be fully characterized.

In this study, we performed a high-throughput, non-targeted metabolomics analysis using liquid chromatography-tandem mass spectrometry (LC–MS/MS) to elucidate the effects of intravitreal anti-VEGF injections on the metabolite profiles in the AH of ME patients.

## Methods

### Study participants and AH samples

A total of 60 patients with ME, including 20 with AMD, 20 with BRVO and 20 with DME, were enrolled in the ophthalmology clinic and ward of the Eye Hospital of Wenzhou Medical University between January 2021 and August 2021. Participants were thoroughly examined and diagnosed by physicians based on clinical manifestations and optical coherence tomography (OCT), optical coherence tomography angiography (OCTA) and/or fluorescein fundus angiography (FFA). Patients with a history of intraocular surgery, trauma or systemic disease were excluded from the study. None of the subjects had a history of diabetes, except for those with DME. Given the high prevalence of hypertension in this population, individuals with a history of hypertension were not excluded from the study.

**Fig. 1:**
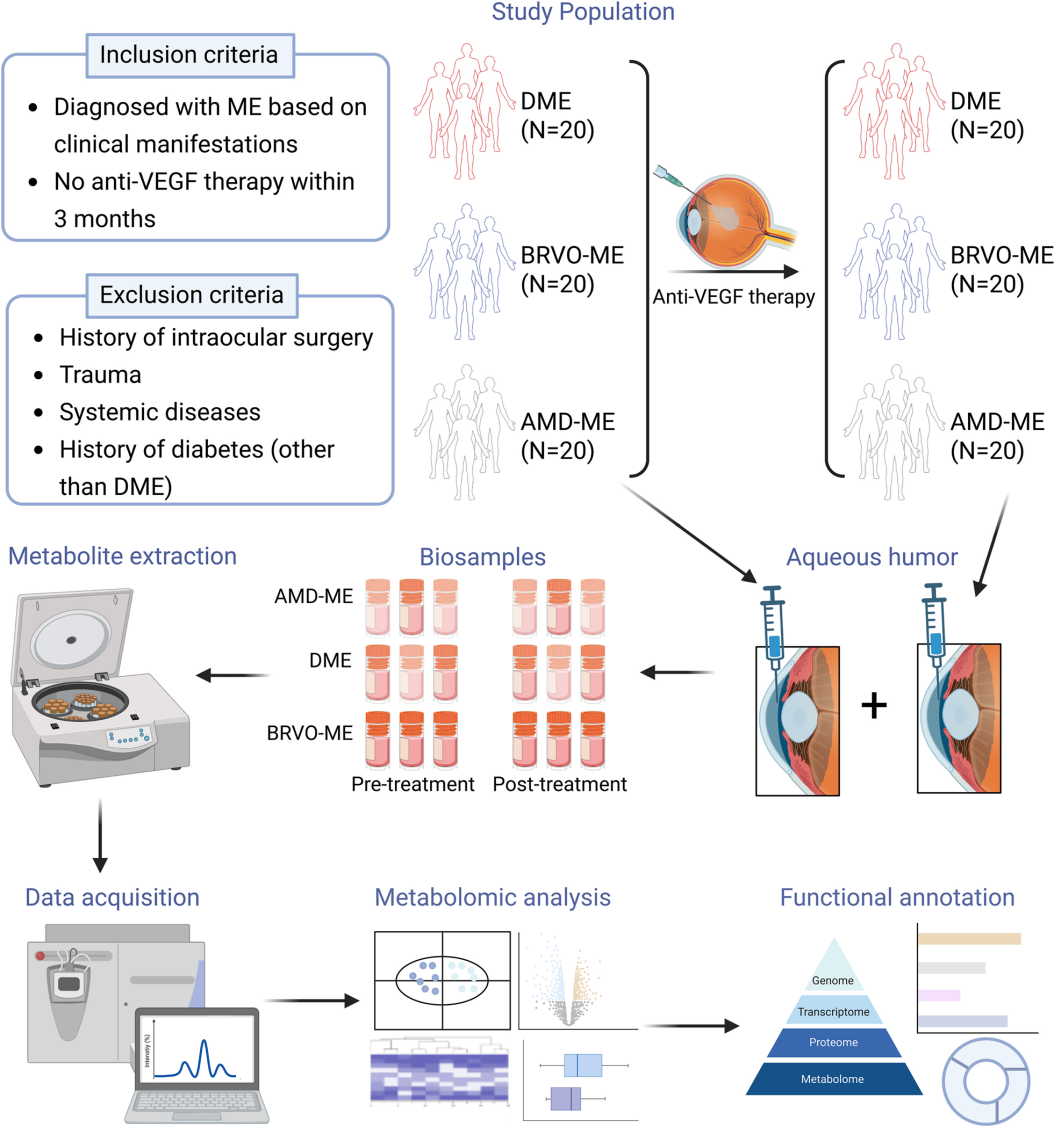
Overview of study design and analytical flow for identifying metabolic features associated with anti-VEGF therapy on macular edema (ME) patients (created with BioRender.com). anti-VEGF, anti-vascular endothelial growth factor; DME, diabetic macular edema; BRVO, branch retinal vein occlusion; AMD, age-related macular degeneration

Of the 60 patients, 87% received their first-ever anti-VEGF injection, while the remaining patients had previously been treated. However, all previously treated patients had not received anti-VEGF treatment for at least three months prior to baseline sampling to ensure that any effects of prior treatments had worn off. Post-treatment samples were collected approximately 30 ± 3 days after intravitreal injection. Additionally, we used two different anti-VEGF agents with uniform doses, with 42/60 patients (20/20 for BRVO, 10/20 for AMD, and 12/20 for DME) receiving ranibizumab, while the other patients receiving conbercept. Patient groups were abbreviated as follows: AA/AF for AMD patients before/after treatment, BA/BF for BRVO patients before/after treatment, and DA/DF for DME patients before/after treatment. Detailed clinical information of the participants is shown in Additional File 2, Table S1, and the overall study workflow is shown in Fig. 1.

A total of 120 AH samples were collected from 60 patients before and after anti-VEGF treatment by standard intravitreal injection. The eye was sterilized according to standard ophthalmic surgical protocols, and approximately 0.1 mL of AH was manually aspirated into a 1.5 mL centrifuge tube. AH samples were stored at −80 °C within 24 h of collection for long-term storage.

This study was approved by the Ethics Committee of the Eye Hospital of Wenzhou Medical University (No. 2021-096-k-80-01). Written informed consent was obtained from all participants before participation in the study.

### LC–MS/MS

Metabolite extraction, quality control (QC) preparation and LC–MS/MS protocols are detailed in the supplementary information (Additional File 1).

### Metabolomics analysis

Orthogonal partial least squares discriminant analysis (OPLS-DA) was used to maximize variation between groups and identify metabolites significantly contributing to the group differences using SIMCA (v.16.0.2, Sartorius Stedim Data Analytics AB, Umea, Sweden). The OPLS-DA model, evaluated by sevenfold cross-validation, was used to assess its predictability and reliability, based on the correlation coefficient (R^2^) and cross-validated R^2^ (Q^2^), which indicate the goodness of fit and predictive ability, respectively. Permutation testing was conducted 200 times to avoid overfitting. Variable importance in projection (VIP) was used to assess the contribution of metabolites to the classification. Differentially expressed metabolites (DEMs) were identified based on a *P* value < 0.05 from paired t-tests and VIP scores ≥ 1. A two-sided t-test with Satterthwaite correction was used to account for unequal variances between groups.

### Enrichment analysis and annotation

Pathway enrichment analysis and metabolite annotation were conducted using MetaboAnalyst (https://www.metaboanalyst.ca/), with metabolites mapped to the Kyoto Encyclopedia of Genes and Genomes (KEGG) and Human Metabolome Database (HMDB) libraries, respectively.

### Statistical analysis

All statistical analyses were performed with the R software environment (v.3.6). Comparisons between different groups were performed using the Student’s t-test. Correlation analysis was conducted to evaluate the relationship between samples using the R package ‘stats’ and ‘corrplot’. Hierarchical clustering was carried out using the R package ‘pheatmap’.

## Results

### Untargeted metabolomics reveals altered metabolic features associated with anti-VEGF therapy

To assess the overall stability and reproducibility of the analytical method, we constructed 15 pooled QC samples from all extracts. These QC samples clustered closely together and were distinctly separated from the subject samples in both negative electrospray ionization (ESI−) and positive electrospray ionization (ESI+) modes, demonstrating high repeatability during the experiment (Additional File 1, Figure S1a, b). Pearson correlation analysis of the ESI− and ESI+ QC samples revealed strong correlations (Figure S1c, d). The coefficient of variation (CV) based on peak area in the pooled QC samples showed that 99% of the metabolites had a CV of less than 30% in both ESI+ and ESI− modes (Additional File 1, Figure S1e, f). These QC measures ensured the high quality of the data obtained in our study.

**Fig. 2:**
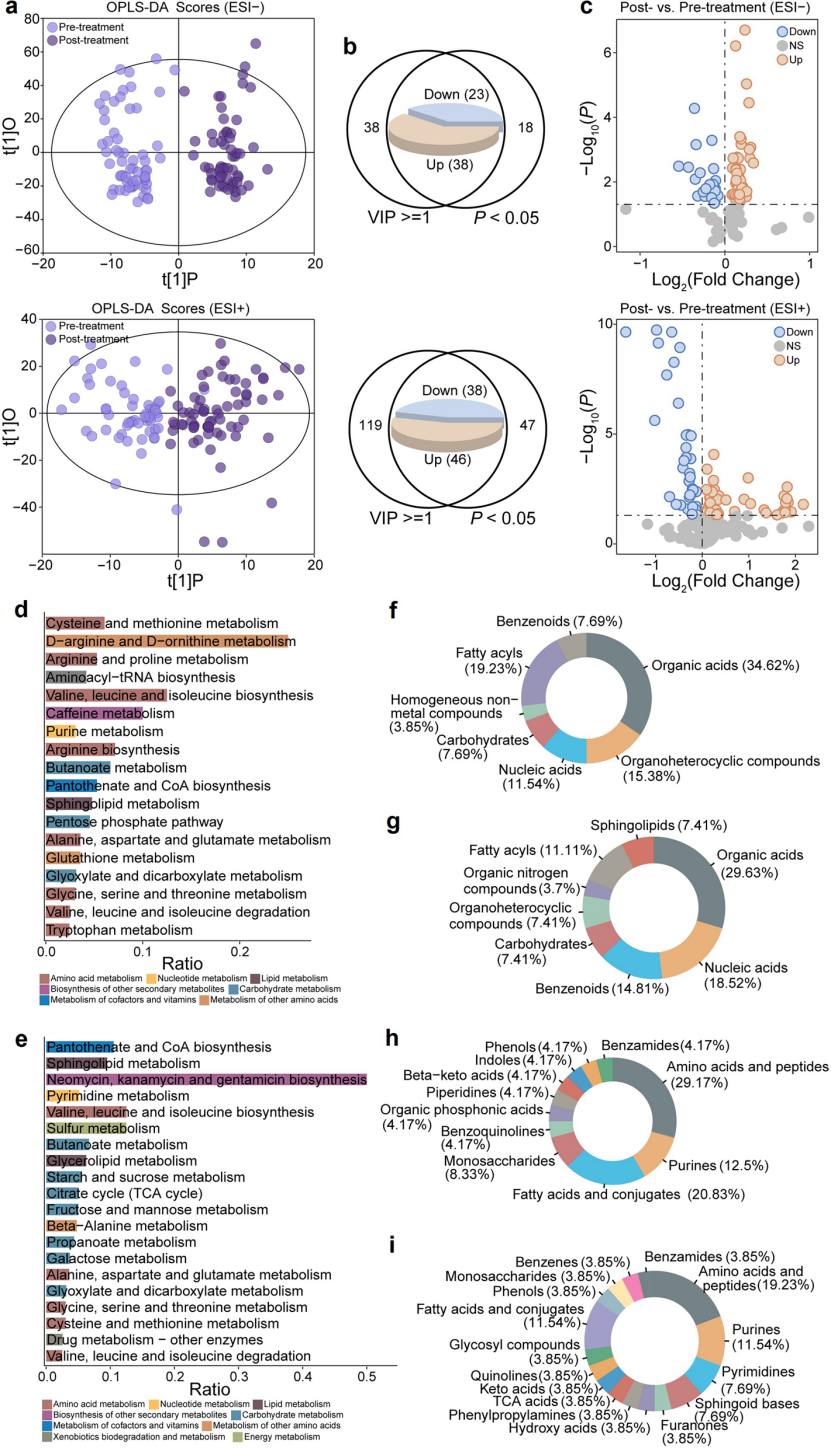
Metabolic profiles and associated pathways in the aqueous humor of patients with macular edema before and after anti-vascular endothelial growth factor (anti-VEGF) therapy. **a** Orthogonal partial least squares discriminant analysis (OPLS-DA) score scatterplots showing metabolic perturbations post-treatment in both negative ion mode (top) and positive ion mode (bottom). **b** Venn diagrams showing the identification of statistically significant metabolites based on variable importance in projection (VIP) ≥ 1 and paired t-test *P* < 0.05. **c** Volcano plot of differentially expressed metabolites (DEMs) comparing pre- and post-treatment samples in both negative ion mode (top) and positive ion mode (bottom). The y-axis represents the log_10_ (*P* value), and the x-axis represents the log_2_ fold change. **d** Enrichment analysis of downregulated metabolites. **e** Enrichment analysis of upregulated metabolites. The x-axis displays the ratio, which is the count of differential metabolites in the pathway/counts of the pathway. The colors indicate the pathway category. **f** Superclass distribution of downregulated metabolites. **g** Superclass distribution of upregulated metabolites. **h** Class distribution of downregulated metabolites. **i** Class distribution of upregulated metabolites. The area reflects the relative fraction of metabolites in each classification, and the various colors represent different Human Metabolome Database (HMDB) categories. ESI−, negative electrospray ionization; ESI+, positive electrospray ionization; tRNA, transfer RNA; CoA, coenzyme A; TCA, tricarboxylic acid

To investigate the molecular impact of anti-VEGF therapy on ME patients, we analyzed metabolomic changes in AH samples collected before and after treatment from 60 ME patients. OPLS-DA results revealed a clear separation between pre- and post-treatment metabolomic profiles in both ESI+ and ESI− modes, which was validated through permutation testing (Fig. 2a, Additional File 1, Figure S2a, b). A total of 61 metabolites in ESI− mode and 84 metabolites in ESI+ mode were identified as significantly different, with VIP scores ≥ 1 and *P* values < 0.05. Among these, 38 metabolites were upregulated and 23 downregulated in ESI− mode, while 46 metabolites were upregulated and 38 downregulated in ESI+ mode post-treatment (Fig. 2b, c).

Hierarchical clustering of the DEMs revealed distinct metabolomic patterns associated with anti-VEGF treatment (Additional File 1, Figure S2c, d). We also incorporated healthy control cohorts to establish biomarker classifications, distinguishing disease-associated negative markers (upregulated in pathological states and downregulated following treatment) from treatment-responsive positive markers (showing the inverse expression pattern). Our findings revealed a consistent expression profile, where positive markers showed elevated expression levels in both post-treatment specimens and healthy controls, while negative markers maintained significantly higher concentrations in disease-state samples (Additional File 1, Figure S3). Further enrichment analysis was conducted to identify altered metabolic pathways. Downregulated DEMs were predominantly associated with amino acid metabolism, particularly cysteine and methionine metabolism (Fig. 2d, e; Additional File 4, Table S3). In contrast, upregulated DEMs were mainly involved in carbohydrate and amino acid metabolism. The association between amino acid metabolism and retinopathy, including ME related to AMD-ME, is well-documented [22]. The superclass distribution of DEMs is shown in Fig. 2f, g. Notably, the majority of downregulated metabolites were classified as organic acids (34.62%) and fatty acyls (19.23%), whereas the most abundant upregulated metabolites were organic acids (29.63%) and nucleic acids (18.52%) (Fig. 2f, g). According to class annotations from the HMDB database, amino acids and peptides, fatty acids and conjugates were the primary components of both upregulated and downregulated metabolites (Fig. 2h, i). These findings suggested that amino acid-related metabolites and metabolic pathways play a crucial role in the repair processes underlying ME.

### Identification of common metabolic features across different etiologies of ME

**Fig. 3:**
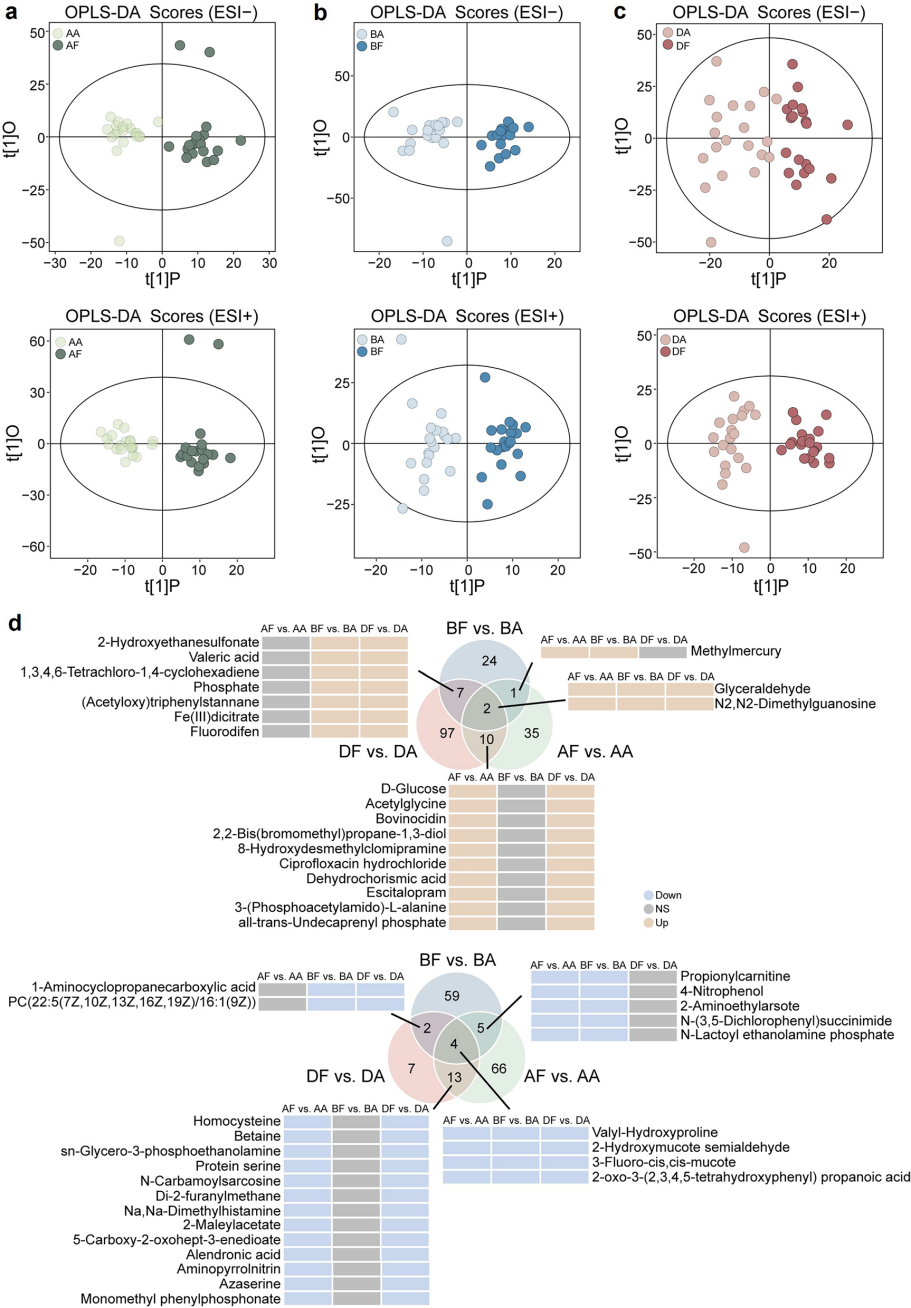
Differentially expressed metabolites between patients with ME due to different retinal diseases. **a-c** OPLS-DA comparing pre-treatment (AA, agate green; BA, light blue; DA, dusty pink) and post-treatment (AF, atrovirens; BF, indigo; DF, magenta) groups in negative ion mode (top) and positive ion mode (bottom). **d** Venn diagrams representing the number of DEMs for each subgroup. Heatmaps represent selected DEMs based on their expression levels in AMD-ME, BRVO-ME or DME. ME, macular edema; OPLS-DA, orthogonal partial least squares discriminant analysis; NS, not significantly up/down-regulated; ESI−, negative electrospray ionization; ESI+, positive electrospray ionization; DEMs, differentially expressed metabolites; AMD, age-related macular degeneration; BRVO, branch retinal vein occlusion; DME, diabetic macular edema; AA, pre-treatment AMD-ME; AF, post-treatment AMD-ME; BA, pre-treatment BRVO-ME; BF, post-treatment BRVO-ME; DA, pre-treatment DME; DF, post-treatment DME

To investigate biochemical perturbations associated with different ME etiologies, we performed OPLS-DA to profile metabolic differences (Fig. 3a–c). OPLS-DA models showed a clear separation between pre- and post-treatment samples in both ion modes for BRVO, AMD, and DME. All models successfully passed a 200-permutation test, confirming the absence of overfitting (Additional File 1, Figure S4). Volcano plots were used to visualize DEMs between pre- and post-treatment samples, indicating significant metabolic dysregulation in patients (Additional File 1, Figure S5a). The relative levels of the significantly altered metabolites are shown in a heatmap (Figure S5b). We then analyzed the DEMs and identified common and disease-specific metabolic alterations in different etiologies of ME. The number of significant metabolites associated with AMD-ME, BRVO-ME, and DME are shown in Fig. 3d. The number of dysregulated metabolites varied significantly across different etiologies of ME: 34 up and 70 down for BRVO-ME, 116 up and 26 down for DME, and 48 up and 88 down for AMD-ME.

**Fig. 4:**
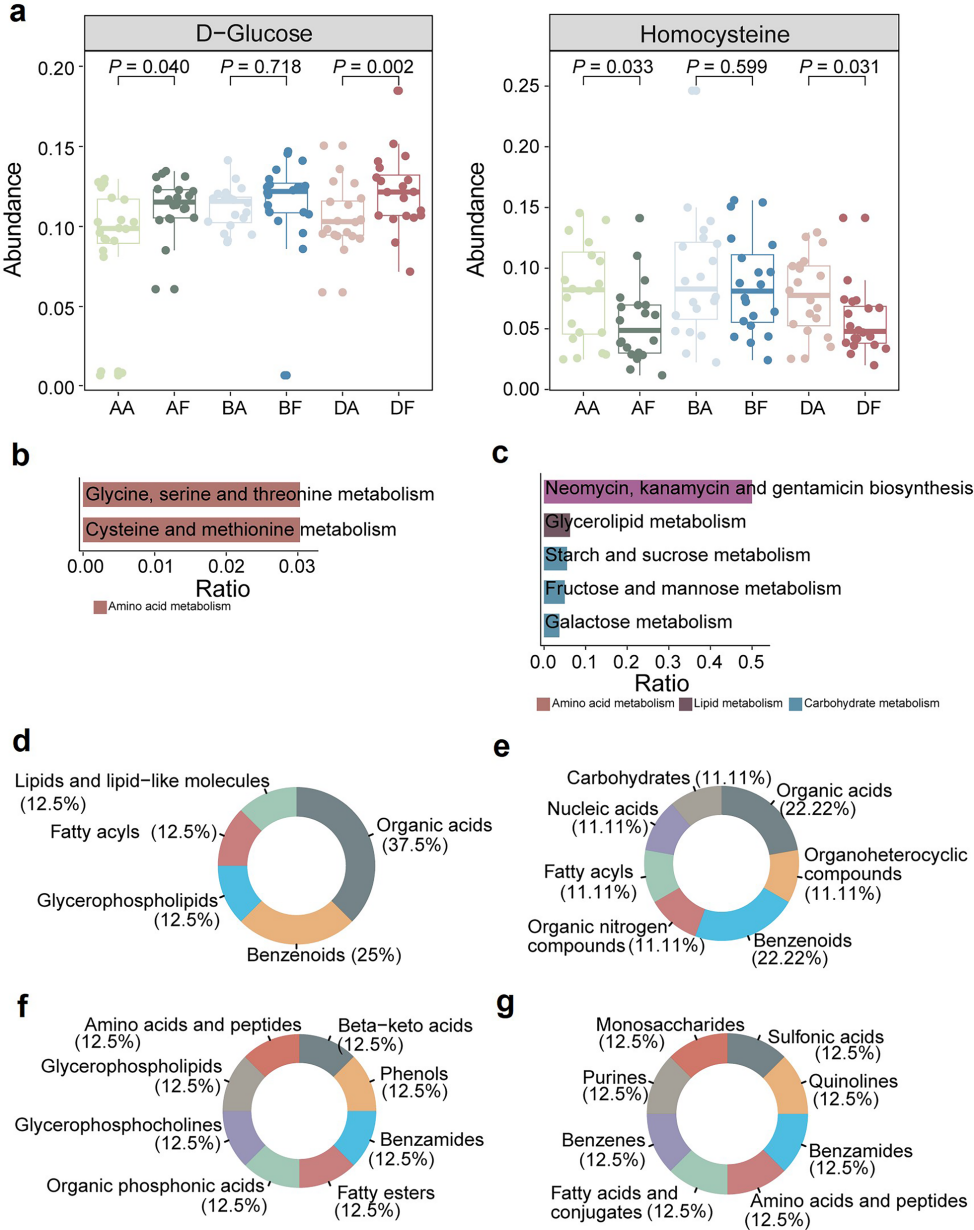
Overview of metabolic analysis of common metabolites across different ME subtypes. **a** Boxplot showing dysregulated metabolic traits between pre-treatment and post-treatment groups. The *P* value was calculated by paired t-test. KEGG enrichment analysis of common (**b**) downregulated and (**c**) upregulated metabolites using MetaboAnalyst. Horizontal bars represent the enrichment ratio, and color indicates the pathway category. Pathways are ranked from top to bottom according to statistical significance. **d** HMDB superclass-level composition of common downregulated metabolites. **e** HMDB superclass-level composition of common upregulated metabolites. **f** HMDB class-level composition of common downregulated metabolites. **g** HMDB class-level composition of common upregulated metabolites. The colors represent different HMDB categories, and the area reflects the relative proportion of metabolites in each classification. ME, macular edema; KEGG, Kyoto Encyclopedia of Genes and Genomes; HMDB, Human Metabolome Database; AMD, age-related macular degeneration; BRVO, branch retinal vein occlusion; DME, diabetic macular edema; AA, pre-treatment AMD-ME; AF, post-treatment AMD-ME; BA, pre-treatment BRVO-ME; BF, post-treatment BRVO-ME; DA, pre-treatment DME; DF, post-treatment DME

Compared to BRVO-ME and AMD-ME, DME showed a higher degree of upregulation and a lower degree of downregulation in DEMs. Compared to other groups, AF vs. AA and DF vs. DA shared the most metabolites, with six metabolites (two upregulated and four downregulated) being common to both diseases. Shared metabolites were defined as those showing differential expression in at least two diseases between pre- and post-treatment states. Among these shared DEMs, glucose was upregulated, and homocysteine was downregulated in post-treatment samples relative to pre-treatment samples. Glucose, a known modulator of angiogenesis, and homocysteine, which inhibits angiogenesis by reducing endothelial cell proliferation [23, 24], may indicate reduced efficacy of anti-VEGF therapy (Fig. 4a). Functional enrichment analysis of ME-shared DEMs using KEGG identified functional categories associated with anti-VEGF treatment, highlighted upregulated carbohydrate and downregulated amino acid metabolism (Fig. 4b, c; Additional File 4, Table S3). Notably, organic acids and benzenoids were the primary superclasses of upregulated and downregulated metabolites, respectively (Fig. 4d, e). Within the class annotations, despite a relatively even distribution of metabolite categories, both upregulated and downregulated metabolites predominantly belonged to benzamides and amino acids and peptides, suggesting a close association with the treatment mechanisms in ME (Fig. 4f, g).

### Characterization of AMD-ME-specific metabolic alterations

**Fig. 5:**
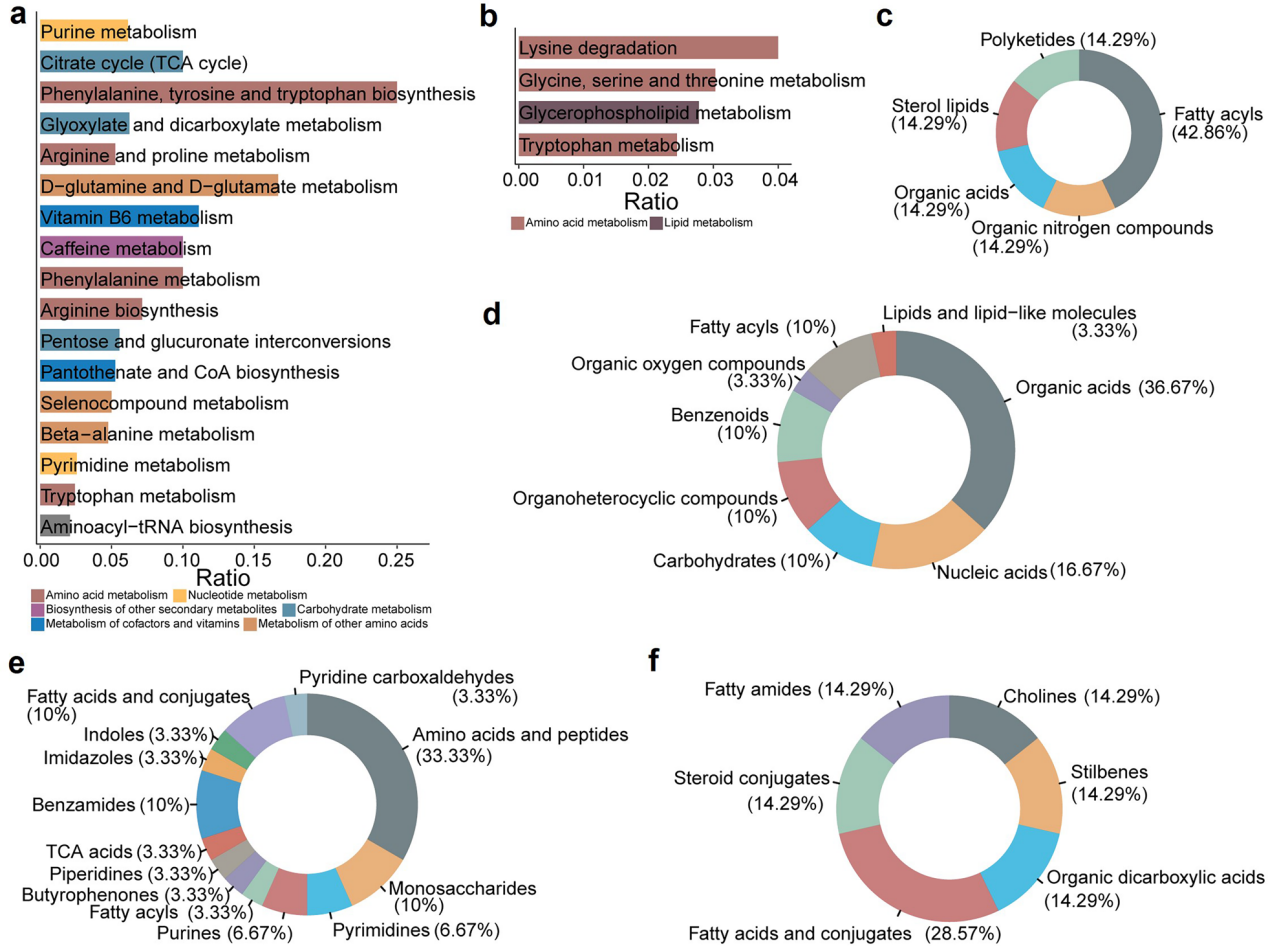
Analysis of unique differential metabolites in AMD-ME patients before and after anti-VEGF therapy. Enrichment analysis of unique (**a**) downregulated and (**b**) upregulated metabolites. The x-axis displays the enrichment ratio, while the color indicates the pathway category. **c** HMDB superclass levels of upregulated metabolites. **d** HMDB superclass levels of downregulated metabolites. **e** HMDB class levels of downregulated metabolites. **f** HMDB class levels of upregulated metabolites. The area reflects the relative proportion of metabolites in each classification, and the colors represent different HMDB categories. AMD, age-related macular degeneration; ME, macular edema; anti-VEGF, anti-vascular endothelial growth factor; HMDB, Human Metabolome Database; tRNA, transfer RNA; CoA, coenzyme A; TCA, tricarboxylic acid

To investigate the disease-specific metabolic effects of anti-VEGF treatment, we focused on pathways where DEMs were exclusive to one of the three diseases. In AMD-ME, 66 metabolites were uniquely downregulated, and 35 were upregulated. Post-treatment, downregulation was observed in pathways such as purine metabolism and the tricarboxylic acid (TCA) cycle (Fig. 5a; Additional File 4, Table S3). Both of these pathways are crucial in neurodegeneration as they provide energy and maintain cellular health. Replenishing TCA cycle metabolites has been shown to aid retinal function preservation [25–27]. A number of amino acid-related pathways, including lysine degradation and glycine, serine, and threonine metabolism, were found to be upregulated (Fig. 5b; Additional File 4, Table S3). These pathways have been identified as potential biomarkers of wet AMD [27]. In terms of superclass annotations, downregulated DEMs primarily consisted of organic acids (36.67%) and nucleic acids (16.67%), whereas fatty acyls represented almost half of the upregulated AMD-ME-specific DEMs (Fig. 5c, d). The class annotation further revealed that amino acids and peptides (33.33%), followed by fatty acids and conjugates (28.75%), constituted the majority of both downregulated and upregulated metabolites (Fig. 5e, f). These findings suggested that amino acid-related metabolites and associated pathways play a significant role in AMD-ME pathophysiology and may have potential implications for therapeutic management.

### Characterization of BRVO-ME-specific metabolic alterations

**Fig. 6:**
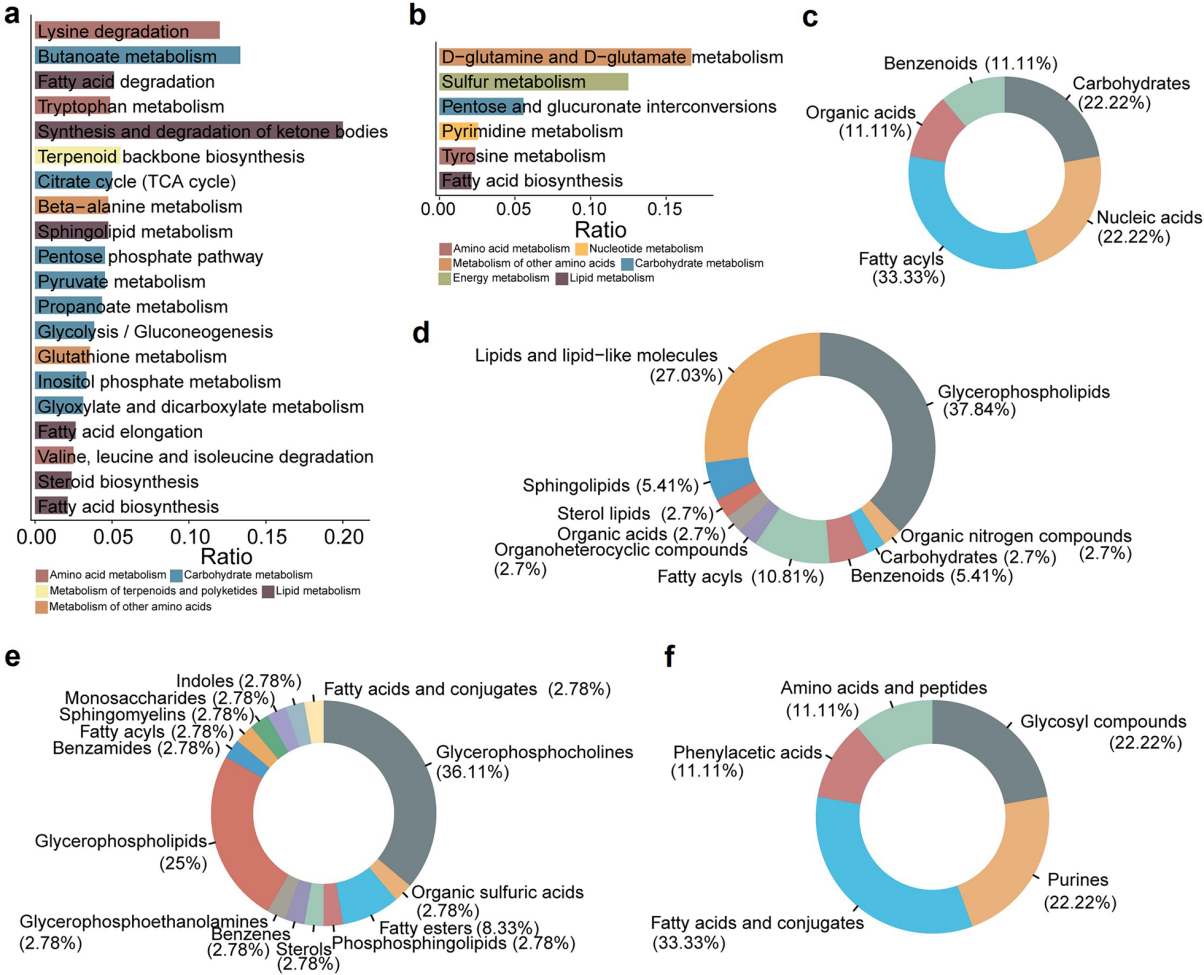
Analysis of unique differential metabolites in BRVO-ME patients before and after anti-VEGF therapy. **a** Enrichment analysis of unique downregulated metabolites. **b** Enrichment analysis of unique upregulated metabolites. The x-axis indicates the enrichment ratio, while the color indicates the pathway category. **c** HMDB superclass levels of upregulated metabolites. **d** HMDB superclass levels of downregulated metabolites. **e** HMDB class levels of downregulated metabolites. **f** HMDB class levels of upregulated metabolites. The area reflects the relative proportion of metabolites in each classification. BRVO, branch retinal vein occlusion; ME, macular edema; anti-VEGF, anti-vascular endothelial growth factor; HMDB, Human Metabolome Database; tRNA, transfer RNA; CoA, coenzyme A; TCA, tricarboxylic acid

In the BRVO-ME subgroup, 59 metabolites were uniquely downregulated, whereas 24 metabolites were upregulated. Several pathways involved in lipid metabolism, particularly those related to fatty acids, were found to be repressed (Fig. 6A; Additional File 4, Table S3), indicating that lipid metabolism abnormalities were responsible for BRVO-ME occurrence. D-glutamine and D-glutamate metabolism and sulfur metabolism, which are associated with nerve damage after cerebral ischemia, were significantly upregulated in post-treatment compared to pre-treatment [28, 29], suggesting their relevance to anti-VEGF treatment of BRVO-ME (Fig. 6b; Additional File 4, Table S3). Among these dysregulated pathways, fatty acid biosynthesis was associated with both upregulated and downregulated DEMs. The superclass annotations indicated that downregulated DEMs mainly consisted of glycerophospholipids, lipids and lipid-like molecules, whereas fatty acyls were predominantly upregulated in BRVO-ME-specific DEMs (Fig. 6c, d). There is substantial evidence that lipid mediators, particularly those derived from glycerophospholipids, play an essential role in angiogenesis [30–32]. Class annotations showed that glycerophosphocholines (36.11%) and glycerophospholipids (25%) were the two most downregulated, whereas fatty acids and conjugates (33.33%) were the most upregulated (Fig. 6e, f). These findings suggested that BRVO-ME is more closely associated with metabolites and pathways related to lipid metabolism.

### Characterization of DME-specific metabolic alterations

**Fig. 7:**
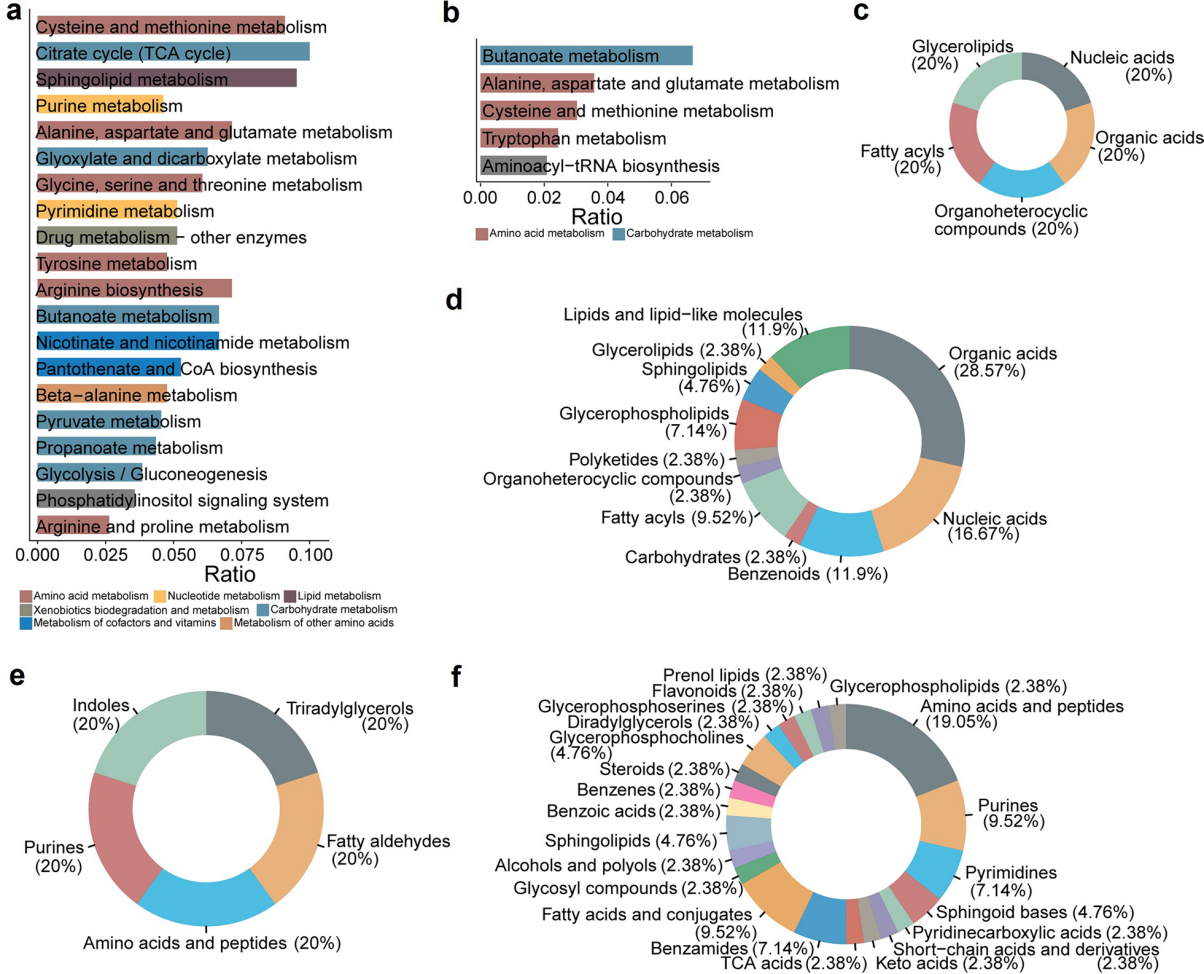
Analysis of unique differential metabolites in DME patients before and after anti-VEGF therapy. **a** Enrichment analysis of unique upregulated metabolites. **b** Enrichment analysis of unique downregulated metabolites. The x-axis shows the enrichment ratio, while the color indicates the pathway category. **c** HMDB superclass levels of downregulated metabolites. **d** HMDB superclass levels of upregulated metabolites. **e** HMDB class levels of downregulated metabolites. **f** HMDB class levels of upregulated metabolites. The area reflects the relative proportion of metabolites in each classification. DME, diabetic macular edema; anti-VEGF, anti-vascular endothelial growth factor; HMDB, Human Metabolome Database; tRNA, transfer RNA; CoA, coenzyme A; TCA, tricarboxylic acid

In the DME group, nine metabolites were uniquely downregulated, while 97 were upregulated. Following anti-VEGF treatment, significant increases were observed in pathways such as cysteine and methionine metabolism, the TCA cycle, sphingolipid metabolism, purine metabolism, and alanine, aspartate, and glutamate pathways (Fig. 7a; Additional File 4, Table S3). In contrast, pathways related to butanoate metabolism and several amino acid pathways showed a decrease (Fig. 7b; Additional File 4, Table S3). The dysregulation of sphingolipid and butanoate metabolism may contribute to metabolic stress in diabetes, which has been implicated in the pathophysiology of DME [33, 34]. Superclass annotations revealed an even distribution of unique downregulated DEMs, whereas unique upregulated DEMs were predominantly composed of organic acids (28.57%) and nucleic acids (16.67%) (Fig. 7c, d). The class categories of downregulated DEMs were also evenly distributed, including amino acid and lipid molecules (Fig. 7e). Amino acids and peptides were the predominant class among upregulated DEMs, followed by purines (Fig. 7f). These results suggested that DME is a complex disease influenced by alterations in both amino acid and carbohydrate metabolism.

## Discussion

Here, we characterized the metabolic alterations induced by intravitreal anti-VEGF injections in the AH of patients with different types of ME using untargeted LC–MS/MS-based metabolomics analysis. Our findings revealed distinct metabolic changes in the AH before and after anti-VEGF therapy across various ME etiologies. Specifically, we identified a number of DEMs and uncovered both common and unique metabolic alterations in different forms of ME. These results highlight the variation in metabolic profiles, which were influenced by the underlying etiology of ME and the therapeutic intervention.

**Fig. 8:**
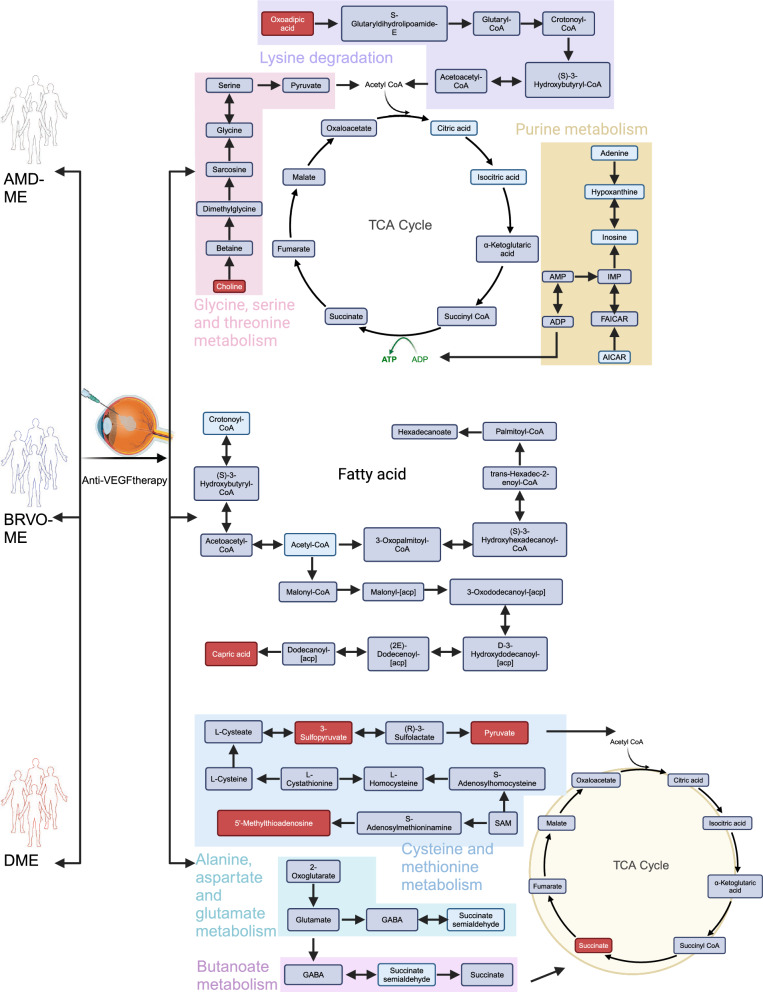
Pivotal metabolic pathways in different subtypes of macular edema groups before and after treatment. The metabolites involved in the dysregulated representative pathways are summarized in detail, and their changes are represented by distinct colors. The red color represents upregulated metabolites, blue represents downregulated metabolites, and gray shows metabolites with no significant difference. ME, macular edema; AMD, age-related macular degeneration; BRVO, branch retinal vein occlusion; DME, diabetic macular edema; CoA, coenzyme A; TCA, tricarboxylic acid; AMP, adenosine 5′-monophosphate; ADP, adenosine 5′-diphosphate; IMP, inosine 5′-monophosphate; ATP, adenosine 5′-triphosphate; FAICAR, 1-(5′-Phosphoribosyl)-5-formamido-4-imidazolecarboxamide; AICAR, 1-(5′-Phosphoribosyl)-5-amino-4-imidazolecarboxamide; anti-VEGF, anti-vascular endothelial growth factor; SAM, s-adenosylmethionine; GABA, 4-aminobutanoate

Our analysis demonstrated distinct patterns in metabolic pathways and associated metabolites, depending on the type of ME. In AMD-ME, amino acid metabolism was significantly affected by anti-VEGF treatment. Conversely, in BRVO-ME, lipid metabolism, particularly fatty acid pathways, showed a more pronounced response to anti-VEGF therapy. In DME, a more complex interaction involving both amino acid and carbohydrate metabolism was observed. Significant changes in metabolites related to amino acids, peptides, fatty acids and their conjugates were observed after treatment in all ME patients. The number of dysregulated metabolites was greater in specific ME subtypes compared to the collective dataset. This could be attributed to the inherent heterogeneity among ME subtypes, which may obscure the metabolic signatures of individual subtypes when combined. By analyzing each subtype independently, we minimized the confounding effects from inter-subtype variation and identified more specific dysregulated metabolites (Fig. 8).

Despite being the standard of care for wet AMD and ME, the impact of anti-VEGF therapy on cellular metabolism, particularly on small molecules known as metabolites, remain underexplored. We observed significant decreases in metabolites related to amino acid metabolism, such as arginine and proline, and in the phenylalanine and arginine biosynthesis pathways. These changes suggest a potential link among these amino acid pathways and the pathophysiology of AMD-ME, including oxidative stress, inflammation, and ischemia. Previous studies have identified significant alterations in amino acid metabolism in the AH and serum of AMD patients, particularly in alanine, aspartate and glutamate pathways [22, 35]. These findings support the hypothesis that specific amino acids might serve as biomarkers for AMD-ME progression and treatment response.

In BRVO-ME, following anti-VEGF treatment, we identified 59 downregulated metabolites and 24 upregulated metabolites. Notably, lipid metabolism, particularly fatty acids, was significantly suppressed. Glycerophosphocholines (36.11%) and glycerophospholipids (25%) were the primary downregulated metabolites. This finding is consistent with previous studies suggesting that dysregulation in lipid metabolism plays a key role in retinal vein occlusion, similar to findings in individuals with ischemic stroke [36, 37]. Furthermore, a metabolomic study on ischemic retinopathy highlighted dysregulation in lipid pathways [38]. This aligns with our findings of suppressed fatty acid metabolism and decreased glycerophospholipid levels in BRVO-ME following anti-VEGF treatment. Phospholipids such as phosphorylcholine, which have known angiogenic effects, may play a role in BRVO-ME progression. While some studies have emphasized amino acid metabolism in BRVO-ME, our study suggests a more pronounced association between anti-VEGF therapy and lipid metabolism. The observed reduction in lipid levels after anti-VEGF treatment may provide new therapeutic targets for managing BRVO-ME.

VEGF is a well-known driver of DR and DME. Our study identified nine downregulated and 97 upregulated metabolites in DME after treatment. We observed significant activity in pathways involving cysteine and methionine metabolism, TCA cycle, sphingolipids, purine and alanine, aspartate and glutamate. These changes suggest that DME patients may undergo complex metabolic reprogramming involving amino acid, lipid, and carbohydrate metabolism. Furthermore, our study also observed a reduction in glutamate levels in DME following anti-VEGF treatment, which may be related to treatment efficacy. Elevated glutamate levels in the retina are known to cause neurotoxicity and overstimulation of N-methyl-D-aspartate receptors (NMDARs), leading to cell damage [39–41]. The observed changes in glutamate metabolism in DME patients may represent a therapeutic avenue to mitigate neurotoxicity and improve treatment outcomes. Our study suggests that anti-VEGF therapy significantly alters the metabolic profiles of ME patients, affecting metabolites involved in amino acid, peptide, fatty acid, and carbohydrate metabolism. These metabolic changes may contribute to the therapeutic effects of the treatment.

There are limitations to our study. First, the small sample size and short follow-up duration may limit the generalizability and depth of our findings. Additionally, as most patients in our cohort exhibited a favorable response to treatment, the identified metabolite alterations may reflect general treatment effects rather than being indicative of response variability. This limitation highlights the need for larger, longer-term studies to determine whether specific metabolite changes predict treatment outcomes. Second, the lack of appropriate animal models that accurately reflect the human ME poses a challenge for mechanistic studies to better elucidate the underlying pathways. Third, in untargeted metabolomics analysis, distinguishing structural isomers and enantiomers of amino metabolites is still a challenge due to their similar chemical structures and physicochemical properties. Finally, we lack independent validation of the metabolic changes identified. Further confirmation using targeted metabolomics or other methods is essential to corroborate our findings and assess their potential clinical significance.

## Conclusions

This study investigated the metabolic changes induced by intravitreal anti-VEGF injection in the AH of patients with ME and compared the metabolic profiles across different ME etiologies using LC–MS/MS-based untargeted metabolomics. Our findings reveal a range of common and etiology-specific metabolic alterations, particularly in carbohydrates, amino acid and lipid metabolism. These findings provide valuable insights into the metabolic processes underlying ME pathogenesis and treatment response.

## Supplementary Information

Additional file 1.

Additional file 2.

Additional file 3.

Additional file 4.

Additional file 5.

## Data Availability

The original LC MS/MS spectrum in ESI− and ESI+ mode during this study are included in Additional File 3, Table S2.

## Abbreviations

AMD: Age-related macular degeneration
AH: Aqueous humor
BRVO: Branch retinal vein occlusion
DEM: Differentially expressed metabolite
DME: Diabetic macular edema
ESI+: Positive electrospray ionization
ESI−: Negative electrospray ionization
LC–MS/MS: Liquid chromatography-tandem mass spectrometry
ME: Macular edema
OPLS-DA: Orthogonal partial least squares discriminant analysis
QC: Quality control
TCA: Tricarboxylic acid
VEGF: Vascular endothelial growth factor
