# Supplementary material for: Metabolomics analysis uncovers metabolic changes and remodeling of anti-VEGF therapy on macular edema
Source: Eye Vis (Lond). 2025 Jul 14;12:28. doi: 10.1186/s40662-025-00444-2 (PMC12257654; doi:10.1186/s40662-025-00444-2)

**Supplementary information**

**1. Supplementary methods**

**Metabolite extraction and quality control preparation**

100 μL of aqueous humor (AH) samples were transferred to Eppendorf (EP) tubes. To each tube, 200 μL of extraction solution (acetonitrile:methanol, 1:1 v/v, with isotopically-labeled internal standard mix) was added, followed by vortexing for 30 seconds, sonication in an ice-water bath for 10 minutes, and incubation at -40 ℃ for 1 hour. Stable isotope-labeled internal standards, such as deuterium (^2H), carbon-13 (^13C) labeled compounds, and L-Leucine as a supplement, were spiked into each sample to correct for variability in sample preparation and ionization efficiency (Table S4). The samples were then centrifuged at 12,000 rpm (RCF=13,800 g, R=8.6 cm) for 15 minutes at 4 ℃. The supernatant obtained was then transferred into a new glass vial for subsequent analysis. For quality control (QC), a sample was prepared by mixing equal volumes of supernatant from each sample.

**Liquid chromatography-tandem mass spectrometry (LC-MS/MS)**

LC-MS/MS analyses were performed on an Ultra-High-Performance Liquid Chromatography (UHPLC) system (Vanquish, Thermo Fisher Scientific), equipped with a UPLC BEH amide column (2.1 mm × 100 mm, 1.7 μm) and coupled to a Q Exactive HFX mass spectrometer (Orbitrap MS, Thermo Fisher Scientific). The mobile phase consisted of 25 mmol/L ammonium acetate and 25 mmol/L ammonia hydroxide in water (pH = 9.75) (A) and acetonitrile (B). The temperature of the autosampler was maintained at 4 ℃, and the injection volume was set at 3 μL. The flow rate was maintained at 0.5 mL/min during the run. The LC gradient commenced with 95% solvent B for 0.5 minutes, followed by a linear decrease to 65% B at 7.0 minutes and 40% B at 8.0 minutes. The gradient was held at 40% B for 1 minute, then rapidly returned to 95% B at 9.1 minutes, holding at this concentration until the conclusion of the run at 12.0 minutes. The total run time was 12 minutes, with the gradient curve set to Curve 5. The mass-to-charge ratio (m/z) scan range was from 70 to 1050. The QE HFX mass spectrometer was used in the information-dependent acquisition (IDA) mode, controlled by the Xcalibur acquisition software (Thermo Fisher Scientific). In IDA mode, the acquisition software continuously monitors and evaluates the full-scan MS spectrum. Peak intensities were normalized to internal standards to account for variability in sample preparation and ionization efficiency. Electrospray ionization (ESI) source conditions were optimized as follows: sheath gas flow rate at 30 arbitrary units (Arb), auxiliary gas flow rate at 25 Arb, capillary temperature at 350 ℃, full MS resolution set to 60,000, MS/MS resolution set to 7,500, collision energy set to 10/30/60 in normalized collision energy (NCE) mode, and spray voltage set to 3.6 kV (positive) and −3.2 kV (negative).

Raw LC-MS/MS data were converted to mzXML format using ProteoWizard software and processed using the R package XCMS for peak detection, extraction, alignment, and integration. Normalization to the total ion current (TIC) for each sample was used to adjust for differences in overall sample load and minor volume variations. An in-house tandem mass spectrometry (MS/MS) database (BiotreeDB) was used for metabolite annotation. The threshold for metabolite annotation was set at a score of 0.3. In cases where multiple metabolites mapped to the same name, the metabolites with the highest MS2 score and the MS1 ppm closer to zero were used.

**2. Supplementary Figures**

**Figure S1. Overview of data quality. a** Principal Component Analysis (PCA) score plot of quality control (QC) samples in negative ion mode. **b** PCA score plot of QC samples in positive ion mode. **c** Pearson correlation analysis between QC samples in negative ion mode. **d** Pearson correlation analysis between QC samples in positive ion mode. The numbers indicate the correlation coefficients, and the lines represent the regression curves. **e, f** Bar plots showing the percentage of metabolites falling within each CV range in **(e)** negative ion mode and **(f)** positive ion mode. The black line indicates the cumulative percentage of metabolites as the CV intervals increase. ESI−, negative electrospray ionization; ESI+, positive electrospray ionization; CV, coefficient of variation.


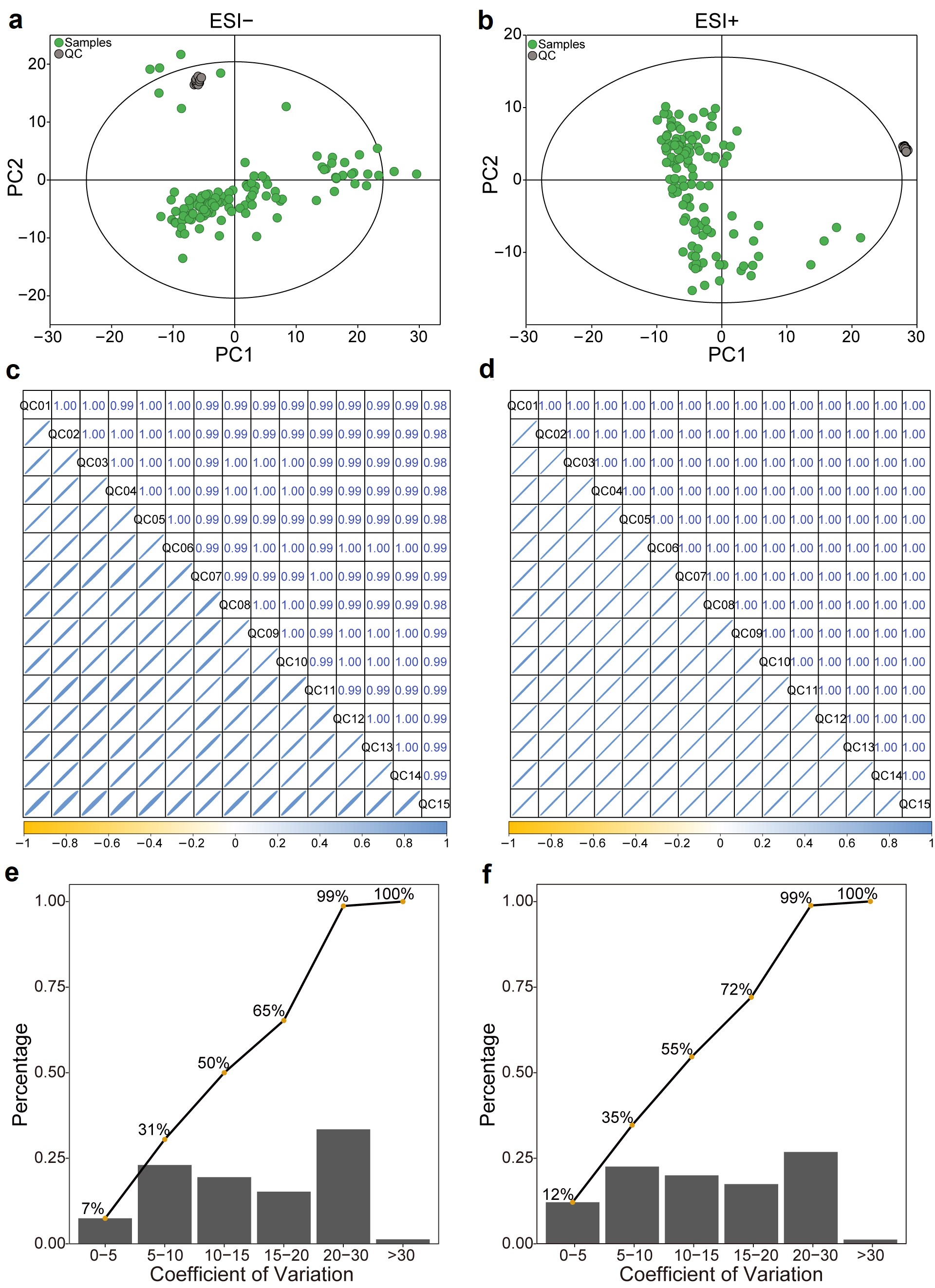


**Figure S2.** **Analysis of metabolites showing significant changes in paired patient samples before and after therapy in patients with macular edema.** **a, b** Permutation test results for the OPLS-DA model. The two points on the right represent the Q2 and R2 values of the actual OPLS-DA model while the two points on the left represent the Q2 and R2 values from 200 permutation tests. **c, d** Heatmap illustrating the abundance of differentially expressed metabolites in negative **(c)** and positive **(d)** ion modes. OPLS-DA, orthogonal partial least squares discriminant analysis; ESI−, negative electrospray ionization; ESI+, positive electrospray ionization.


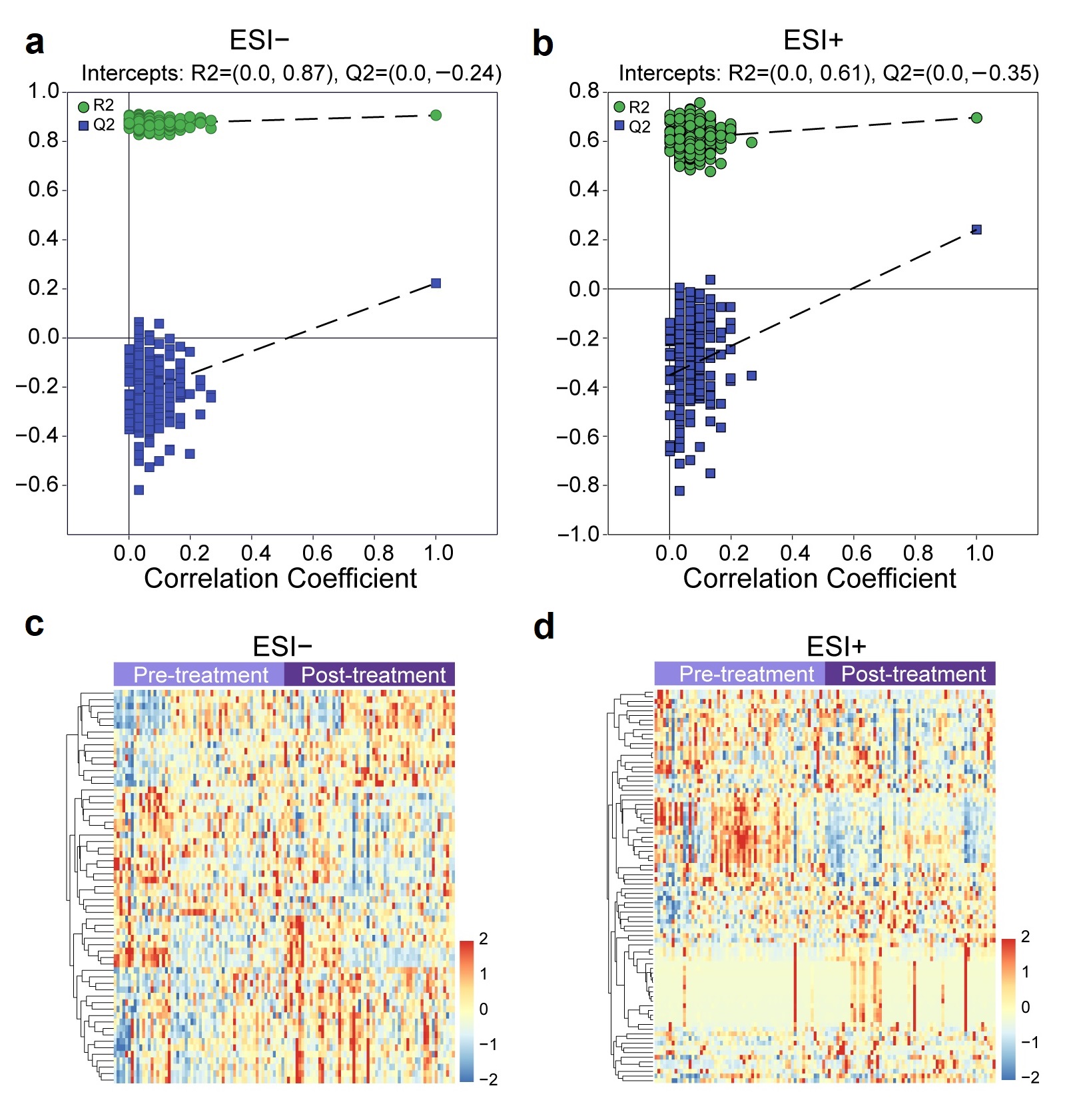


**Figure S3.** **Analysis of metabolites that showed significant changes in paired patient samples before and after therapy in patients with macular edema or between disease and normal samples.** Metabolites that are upregulated in the disease group or downregulated after treatment are called negative markers and vice versa are called positive markers.


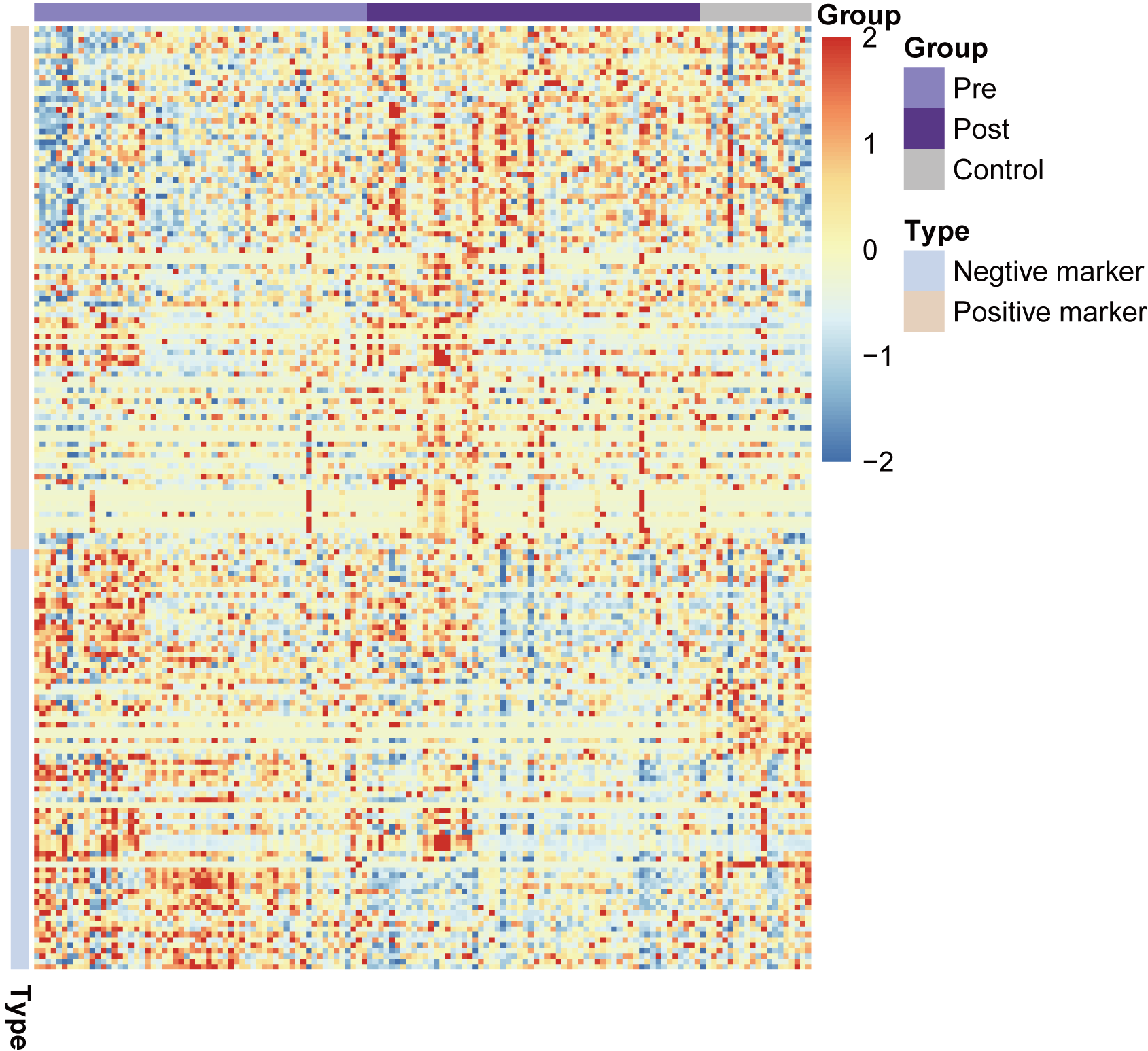


**Figure S4.** **Validation of the OPLS-DA model using a permutation test with 200 permutations.** **a** AMD-ME. **b** BRVO-ME. **c** DME. OPLS-DA, orthogonal partial least squares discriminant analysis; ME, macular edema; AMD, age-related macular degeneration; BRVO, branch retinal vein occlusion; DME, diabetic macular edema; AA, pre-treatment AMD-ME; AF, post-treatment AMD-ME; BA, pre-treatment BRVO-ME; BF, post-treatment BRVO-ME; DA, pre-treatment DME; DF, post-
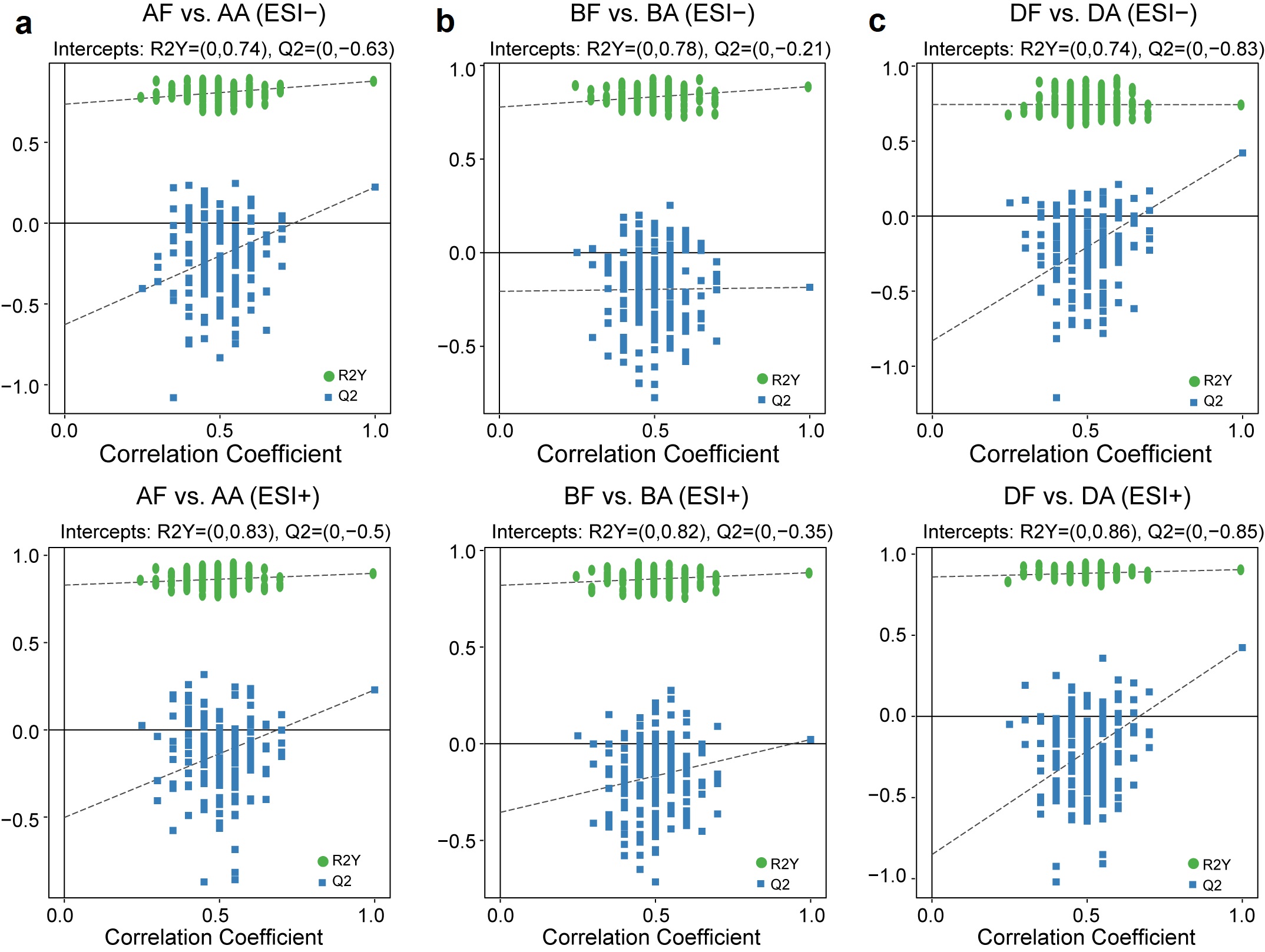
treatment DME.

**Figure S5. Frequency of DEMs across three groups**. **a** Volcano plot. **b** Hierarchical clustering of patients with ME due to different retinal diseases and matched groups. ESI−, negative electrospray ionization; ESI+, positive electrospray ionization; DEMs; differentially expressed metabolites; ME, macular edema; AMD, age-related macular degeneration; BRVO, branch retinal vein occlusion; DME, diabetic macular edema; AA, pre-treatment AMD-ME; AF, post-treatment AMD-ME; BA, pre-treatment BRVO-ME; BF, post-treatment BRVO-ME; DA, pre-treatment DME; DF, post-treatment DME.


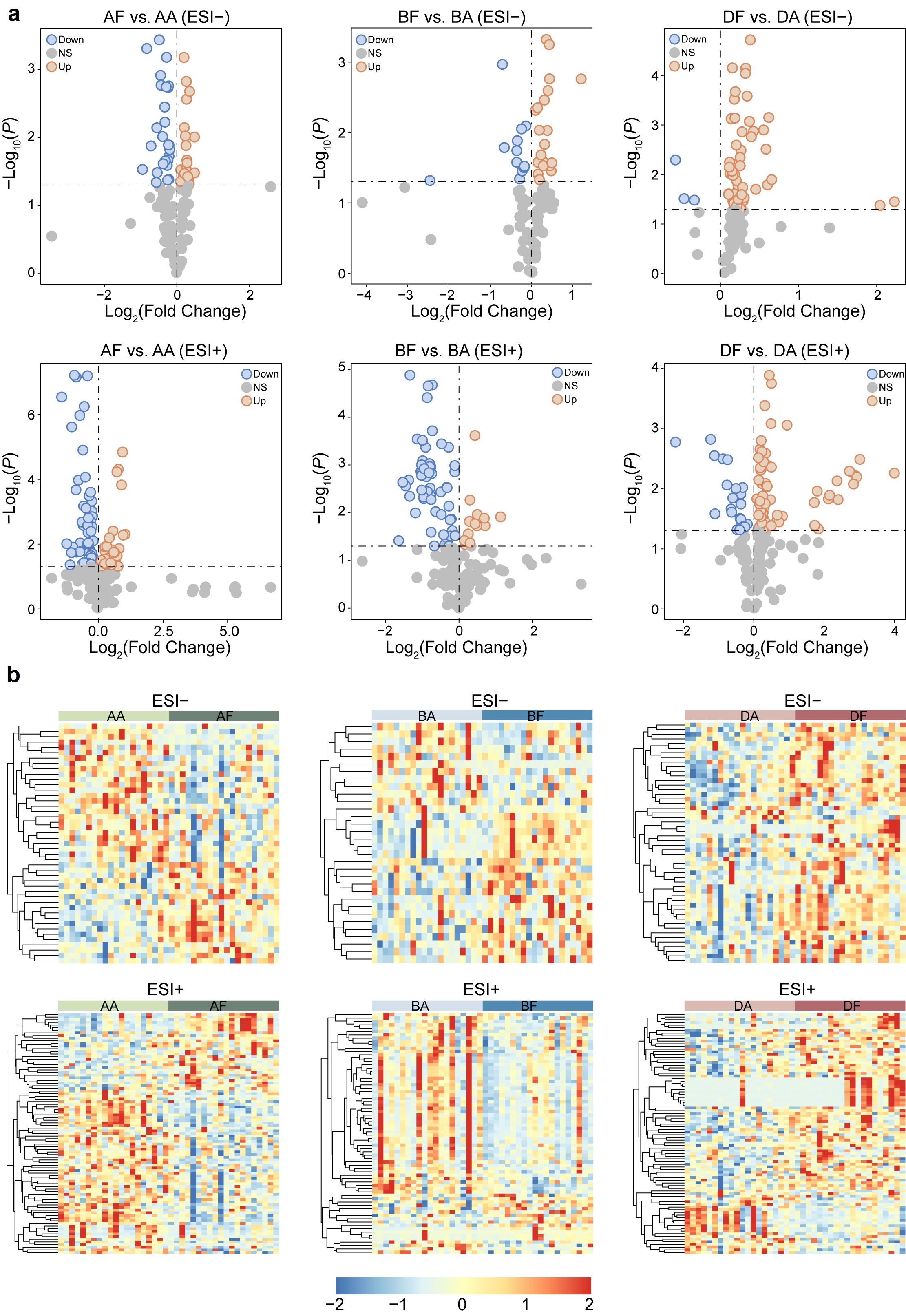

Supplement: Supplementary file 1 — Additional file 1. [file 40662_2025_444_MOESM1_ESM.doc]
