# Supplementary material for: Metabolomics analysis uncovers metabolic changes and remodeling of anti-VEGF therapy on macular edema
Source: Eye Vis (Lond). 2025 Jul 14;12:28. doi: 10.1186/s40662-025-00444-2 (PMC12257654; doi:10.1186/s40662-025-00444-2)
Supplement: Supplementary file 2 — Additional file 2. [file 40662_2025_444_MOESM2_ESM.docx]

**Supplementary Table S1. Clinical characteristics of ME patients in this study.**

| **Characteristics** | **AMD-ME** | **BRVO-ME** | **DME** |
| --- | --- | --- | --- |
| AH samples (cases) | 40 (20) | 40 (20) | 40 (20) |
| Male/female | 11/9 | 11/9 | 8/12 |
| Age (years, mean ± SD) | 64.4 ± 6.5 | 61.7 ± 11.2 | 56.1 ± 9.2 |
| CST before/after treatment (µm, median [min, max]) | 433 µm [177, 737]/  261.5 µm [139, 485] | 562.5 µm [216, 1053]/  233 µm [139, 581] | 487 µm [203, 955]/  281 µm [179, 645] |
| Hypertension | 7 (35%) | 8 (40%) | 7 (35%) |
| Diabetes | 0 (0%) | 0 (0%) | 20 (100%) |
| Cataract | 80% | 65% | 75% |
| Eye surgery (within three months) | 0% | 0% | 0% |
| Vitreous hemorrhage | 0% | 0% | 0% |

AMD = age-related macular degeneration; ME = macular edema; BRVO = branch retinal vein occlusion; DME = diabetic macular edema; VEGF = vascular endothelial growth factor.
