# Supplementary material for: Metabolomics analysis uncovers metabolic changes and remodeling of anti-VEGF therapy on macular edema
Source: Eye Vis (Lond). 2025 Jul 14;12:28. doi: 10.1186/s40662-025-00444-2 (PMC12257654; doi:10.1186/s40662-025-00444-2)
Supplement: Supplementary file 5 — Additional file 5. [file 40662_2025_444_MOESM5_ESM.docx]

**Supplementary Table S4. Stable isotope-labeled internal standards**

| **Compound** | **ESI+ (m/z)** | **ESI− (m/z)** | **CAS** |
| --- | --- | --- | --- |
| L-Leucine-5,5,5-d3 | 135.1207 | – | 87828-86-2 |
| 4-Aminobutyric acid-2,2,3,3,4,4-d6 (GABA-d6) | 110.1083 | – | 70607-85-1 |
| Acetylcholine-d9 (N,N,N-Trimethyl-d9) | 155.174 | – | 344298-95-9 |
| L-Leucine | – | 133.1062 | 87828-86-2 |
| Octanoic acid-d15 | – | 158.2019 | 69974-55-6 |
| o-Chlorophenylacetic acid-ring-13C6 | – | 142.0605 | 335081-06-6 |

CAS = chemical abstracts service.
